# Microscopic Mechanism Study on Gas–Crude-Oil Interactions During the CO_2_ Flooding Process in Water-Bearing Reservoirs

**DOI:** 10.3390/ijms26136402

**Published:** 2025-07-03

**Authors:** Wei Xia, Yu-Bo Wang, Jiang-Tao Wu, Tao Zhang, Liang Gong, Chuan-Yong Zhu

**Affiliations:** 1 Key Laboratory of Thermo-Fluid Science and Engineering, Ministry of Education, School of Energy and Power Engineering, Xi’an Jiaotong University, Xi’an 710049, China; weixia83@outlook.com (W.X.); jtwu@mail.xjtu.edu.cn (J.-T.W.); 2 College of New Energy, China University of Petroleum (East China), Qingdao 266580, China; z24150119@s.upc.edu.cn (Y.-B.W.); tao.zhang@upc.edu.cn (T.Z.); lgong@upc.edu.cn (L.G.)

**Keywords:** crude oil, CO_2_, water film, molecular simulation

## Abstract

The impact of water on CO_2_ sequestration and enhanced oil recovery processes is significant. In this study, a CO_2_–water-film–crude-oil–rock molecular system was established. Then, the influence of water-film thickness on the dissolution and dispersion of CO_2_ and crude oil under different temperature and pressure scenarios was examined through molecular dynamics simulations. The results indicate that water films hinder CO_2_ diffusion into the oil, reducing its ability to lower oil density. When the thickness of the water film increases from 0 nm to 3 nm, the oil density increases by 86.9%, and the average diffusion coefficient of oil decreases by 72.30%. Increasing the temperature enhances CO_2_–oil interactions, promoting CO_2_ and water diffusion into oil, thereby reducing oil density. Under conditions of a 2 nm water film and 10 MPa pressure, increasing the temperature from 100 °C to 300 °C results in a decrease of approximately 32.1% in the oil density. Pressure also promotes oil and water-film density reduction, but its effect is less significant compared to temperature. These results elucidate the function of the water film in CO_2_-EOR processes and its impact on CO_2_ dissolution and diffusion in water-bearing reservoirs.

## 1. Introduction

As the primary source of energy, oil remains a crucial driver of advancements across various sectors [1,2]. Increasing oil production is of great significance for meeting the global energy demand and ensuring energy security [3]. However, following decades of development through water flooding techniques, many oil reservoirs have entered a low-permeability and high-water-content stage, posing severe challenges to crude-oil extraction [4,5]. CO_2_ flooding can cause crude-oil expansion, promote the release of hydrocarbons, and reduce crude-oil viscosity, thereby improving recovery efficiency [6,7]. In addition, injecting CO_2_ into the formation has the potential to substantially lower the release of greenhouse gases into the atmosphere [8]. Therefore, carrying out research on CO_2_-enhanced oil recovery technology in water-containing reservoirs holds considerable importance for advancing environmental and energy extraction goals [8,9,10].

Zhang et al. [11] investigated oil diffusion behavior in various rock pores in the presence of water. They reported that the hydrophobic nature of organic surfaces combined with the strong hydrogen bonding among water molecules causes water to tend to gather at the center of graphite-like pores, which markedly inhibits oil molecular diffusion. Yang et al. [12] studied the adsorptive ability of water and octane on the pore surfaces of different rocks. They found that the adsorption energy of graphite and quartz pores for octane considerably exceeds that for water, and the graphite–octane interaction is stronger. However, the above study did not involve evaluation of the effect of CO_2_ on crude oil. Lu et al. [13] conducted MD simulations to explore the oil displacement by CO_2_ in water-cut dead-end nanopores. They revealed that oil displacement initiates only after the water-film rupture, which is driven by CO_2_ dissolution in water and hydrogen bonding between water and rock, disrupting the water molecular network. Zhao et al. [14] studied CO_2_-enhanced oil mobility in shale inorganic nanopores and reported that the presence of water significantly influences CO_2_–oil interactions. Their results demonstrated that an increasing CO_2_ content reduces the oil–water interfacial tension and promotes CO_2_ accumulation at the interface, which enhances the oil mobility and recovery potential under complex reservoir conditions. Liu et al. [15] examined the impact of CO_2_ on the water–decane interface and found that there is a strong interaction between CO_2_ and water, and between CO_2_ and crude oil. They pointed out that CO_2_ has the ability to lower the water–oil interfacial tension and cause molecular enrichment at the water–decane interface. Luan et al. [16] carried out extensive molecular dynamics (MD) modeling of the displacement of residual oil within dead-end pores under a CO_2_ atmosphere in water-saturated layers. They found that when CO_2_ enters the water layer, it causes the breakage of hydrogen bonds, forming a collapsed pore that serves as a passage for CO_2_ to enter the pore space.

Although the existing literature provides an important theoretical basis for CO_2_ flooding, there are still some key gaps in its microscopic mechanism. Previous studies have explored the inhibitory effect of a water film on CO_2_ displacement and the possibility of CO_2_ penetrating the water film; however, most of them have been limited to the description of phenomena rather than systematically investigating the underlying competitive mechanism. It is particularly noteworthy that there is still a lack of quantitative analysis of how the promoting effect of thermodynamic conditions (temperature and pressure) interacts with the hindering effect of the water film and how this coupling effect affects the displacement process.

To this end, this paper constructs a molecular model of the CO_2_–water-film–crude-oil–rock system based on the four-component composition of crude oil (saturates, aromatics, resins, and asphaltenes) and systematically analyzes the above interactions. This paper first elaborates on the mechanism of water-film thickness as the main inhibitory factor and then deeply examines how temperature and pressure, as the key factors, affect the mass transfer of CO_2_ in the presence of the water film, thereby revealing the dynamic equilibrium relationship between these effects. This work aims to provide an important mechanistic framework for optimizing the operating parameters of CO_2_ flooding in high-water-content reservoirs.

## 2. Results and Discussion

### 2.1. Micro-Mechanism of Gas and Crude-Oil Dissolution

#### 2.1.1. Effect of Water-Film Thickness on Density Distribution

**Figure 1:**
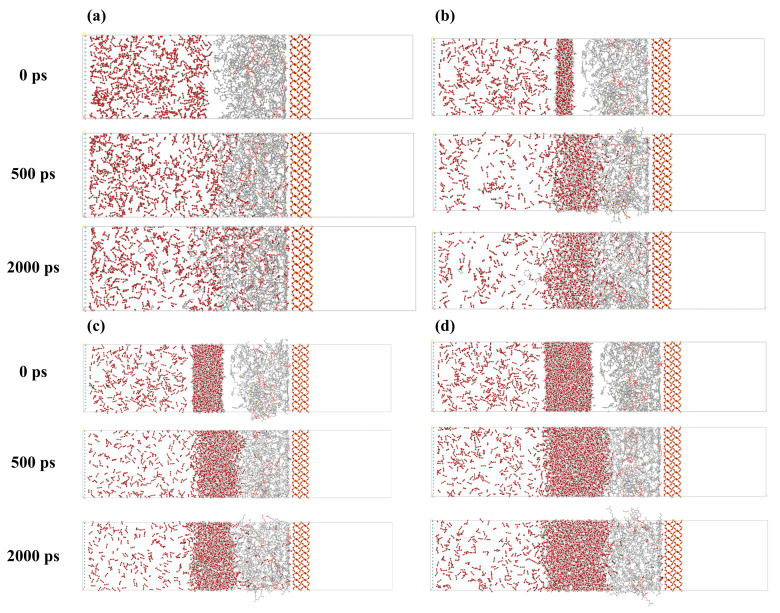
Molecular dynamics simulation of system configuration snapshots under different water-film thicknesses: (**a**) without water film; (**b**) 1 nm water film; (**c**) 2 nm water film; (**d**) 3 nm water film.

Figure 1 depicts a snapshot of the gas–water–oil–rock model over time, where Figure 1a–d correspond to the states of no water film, a 1 nm water film, a 2 nm water film, and a 3 nm water film, respectively. These results show that the thickness of the water film has a significant effect on the spatial distribution of molecules in the system. As can be seen from Figure 1a, in the absence of a water film, CO_2_ molecules can freely penetrate the oil layer and form close contact with the oil phase. This indicates that under the condition of no water film, the interaction between CO_2_ and crude oil is particularly significant, which may lead to a significant decrease in the density of the oil layer and an increase in its diffusion capacity. When the thickness of the water film increases to 1 nm (as shown in Figure 1b), the water film begins to form a water interface, which becomes an obstacle to the penetration of CO_2_ molecules. However, it is worth noting that some CO_2_ molecules can still break through the barrier of the water film and invade the crude oil. As the thickness of the water film increases further (as shown in Figure 1c,d), the inhibitory effect of the water film becomes more significant, and the water film significantly limits the migration of CO_2_ molecules. Therefore, most CO_2_ molecules cannot break through the water film barrier and enter the oil layer.

**Figure 2:**
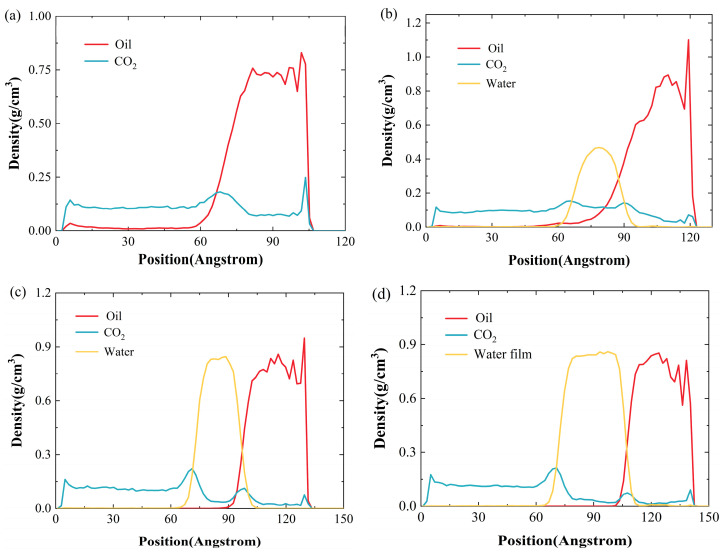
Density profiles of individual components at different water-film thicknesses: (**a**) without water film; (**b**) 1 nm water film; (**c**) 2 nm water film; (**d**) 3 nm water film.

Figure 2 shows the density distributions of various components near the quartz wall at 2000 ps, where Figure 2a illustrates the system without a water film, and Figure 2b–d show the density profiles of individual components across various water-film thicknesses. It is clear that, in all cases, CO_2_ establishes an adsorption layer on the rock surface, with the peak density of this layer progressively diminishing with increasing water-film thickness. Specifically, when the water-film thickness is 1 nm, the CO_2_ adsorption layer density can reach 0.1 g/cm^3^; when the thickness is 2 nm, the density drops to 0.095 g/cm^3^; and when the thickness is 3 nm, it further decreases to 0.079 g/cm^3^, corresponding to 80%, 76%, and 68% of the adsorption density without a water film, respectively. In addition, compared with the water-free film system, the CO_2_ density inside the oil in the water-containing system is significantly reduced. This effect can be ascribed to the water film functioning as a barrier layer, which obstructs the migration of CO_2_ into the oil, leading to a reduction in gas density within the oil and thereby diminishing the adsorption capacity of CO_2_ on the pore wall surface.

As is seen in Figure 2b–d, CO_2_ molecules are also significantly enriched at the water–oil interface, indicating that the water film itself also has a certain adsorption capacity for CO_2_. By comparing Figure 2a–d, it can be further found that as the water-film thickness grows, the density of the crude oil shows a significant upward trend. This is primarily because thicker water films hinder the entry of CO_2_ molecules into the oil. Compared to a scenario lacking a water film, the mean oil density increases by 4.8%, 12.3%, and 86.9% as the water layer thicknesses reach 1, 2, and 3 nanometers, respectively.

In order to study the effect of the water film on the CO_2_–crude-oil interaction, we monitored the crude-oil density and investigated the limiting effect of water-film thickness on the crude-oil expansion effect. As is shown in Figure 2, with the increase in water-film thickness, the crude-oil density showed an upward trend, which directly reflected the suppression of the crude-oil-expansion effect. In the system without a water film, CO_2_ can be fully dissolved in the oil phase, causing the oil phase to expand significantly, resulting in a significant reduction in its density. However, the presence of a water film seriously hinders the transfer process of CO_2_ to the oil phase. Since CO_2_ cannot fully diffuse into the oil phase, the expansion effect of crude oil is significantly suppressed, thereby maintaining a high density level. This result profoundly reveals the key role of water film in regulating the behavior of the oil phase, that is, the water film not only limits the molecular diffusion of CO_2_ but also inhibits the expansion of the oil phase, preventing it from transforming to a favorable state of low density. This mechanism clearly explains the root cause of the negative impact of a water film on CO_2_-EOR recovery efficiency from a quantitative perspective.

#### 2.1.2. Influence of Water-Film Thickness on the Microscopic Interaction Mechanism of CO_2_ and Crude Oil

The radial distribution function (RDF), diffusion coefficient, and interaction energy can reveal the spatial arrangement, diffusion behavior, and binding strength between molecules. Therefore, to further explore the impact of the water layer on the CO_2_–crude–oil interaction, this study examined the radial distribution function, oil diffusion coefficient, and interaction energy between different components under different water-film thicknesses. The RDF represents the ratio of the local density of the region at a given distance to the average density. It reflects the change in particle density as a function of distance. The radial distribution function can be calculated as follows [17]:

(1) $$g(r)=\frac{\rho_{r}}{\rho_{0}}$$

where *ρ*_*r*_ indicates the distribution density of particle B relative to its distance from particle A, and *ρ*_0_ indicates the mean density of particle B in the system.

**Figure 3:**
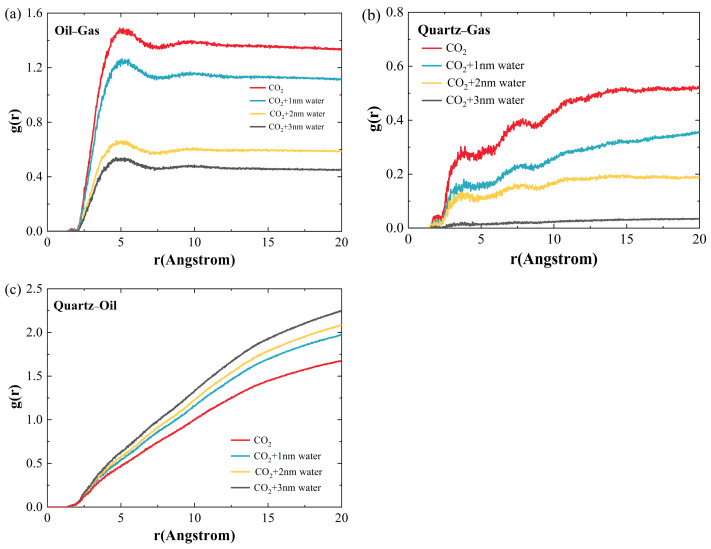
Radial distribution functions (RDFs) between different components and their relationship with water: (**a**) oil–gas; (**b**) quartz–gas; (**c**) quartz–oil.

Figure 3 shows the RDFs between different molecules. As is illustrated in Figure 3a, the RDFs between crude oil and CO_2_ reach a peak at approximately 5.1 Å. As the water film becomes thicker, the RDFs between crude oil and gas gradually decrease, suggesting a reduction in the number of CO_2_ molecules near the crude oil. This is mainly because, with an increasing water-film thickness, it becomes more difficult for CO_2_ molecules difficult to enter into the crude oil. Similarly, as the water-film thickness increases, the RDFs between the quartz and CO_2_ molecules also gradually decrease, indicating a reduced probability of CO_2_ molecules near the quartz surface, as depicted in Figure 3b. Figure 3c plots the RDFs between quartz and crude-oil molecules. It is found that with the thickness of the water film increasing, this RDF increases. The primary reason is that, as the thickness of the water film increases, it becomes more difficult for CO_2_ molecules to enter the crude oil, which leads to a lesser reduction in crude-oil density, thus increasing the probability of oil molecules being near the quartz wall.

The diffusion coefficient reflects the dynamical properties of molecules; the greater the diffusion coefficient, the stronger the molecular activity, which leads to decreased viscosity of the material. The diffusion coefficient of a molecule can be determined by analyzing its mean square displacement (*MSD*). The *MSD* refers to the average of the squared displacement of the molecule from its initial position after time *t* [18]:

(2) $$MSD=\frac{1}{N}\sum_{i=1}^{n}{\left|r_{i}\left(t\right)-r_{i}\left(0\right)\right|}^{2}$$

where *r*_*i*_(*t*) represents the position coordinates of particle *i* at time *t*. The diffusion coefficient can be obtained using the following formula:

(3) $$D=\frac{1}{6N}\underset{m\to \infty }{\lim}\frac{1}{m\cdot \Delta t}\langle \sum_{i=1}^{N}{\left[\vec{r_{i}}\left(t+m\cdot \Delta t\right)-\vec{r_{i}}\left(t\right)\right]}^{2}\rangle$$

**Figure 4:**
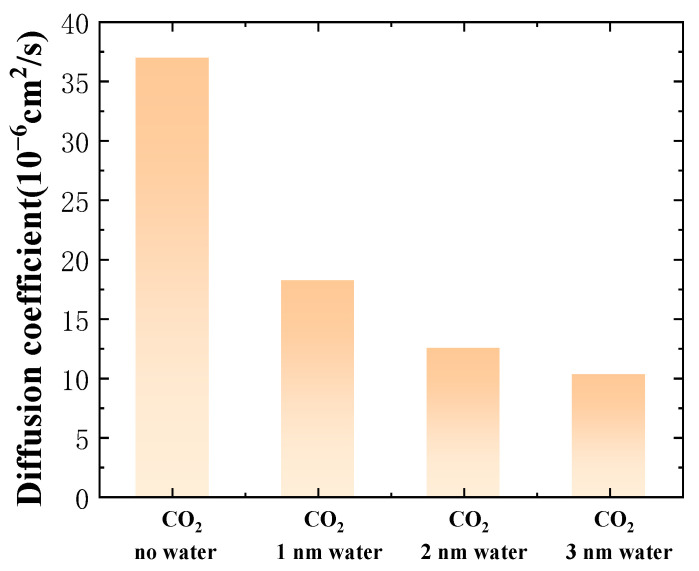
Diffusion characteristics of crude oil at different water-film thicknesses.

Figure 4 shows the diffusion coefficient of oil molecules at various water-film thicknesses. As can be seen in this figure, the diffusion coefficient of crude oil diminishes as the water-film thickness increases. Compared to the system without a water film, the diffusion coefficient of oil decreases by 50.81%, 66.24%, and 72.30% when the water-film thickness is 1 nm, 2 nm, and 3 nm, respectively. This is primarily because CO_2_ itself possesses the capacity to lower the viscosity of crude oil, thereby promoting an increase in the crude-oil diffusion coefficient. However, as the water film becomes thicker, the amount of CO_2_ entering the crude oil significantly decreases (see Figure 4), which impedes the decrease in crude-oil viscosity.

The interaction energy reflects the binding strength between molecules. When values are negative, they indicate attraction; positive values suggest repulsive interactions. The greater the magnitude of the interaction energy, the stronger the binding strength [19]. The interaction energy can be obtained as

(4) $$E_{A-B}=E_{\mathrm{Total}}-(E_{A}+E_{B})$$

where *E*_A−B_ reflects the energy resulting from the interaction of A and B. *E*_Total_ represents the overall interaction energy, and *E*_A_ and *E*_B_ represent the interaction energies between A and B, individually.

**Figure 5:**
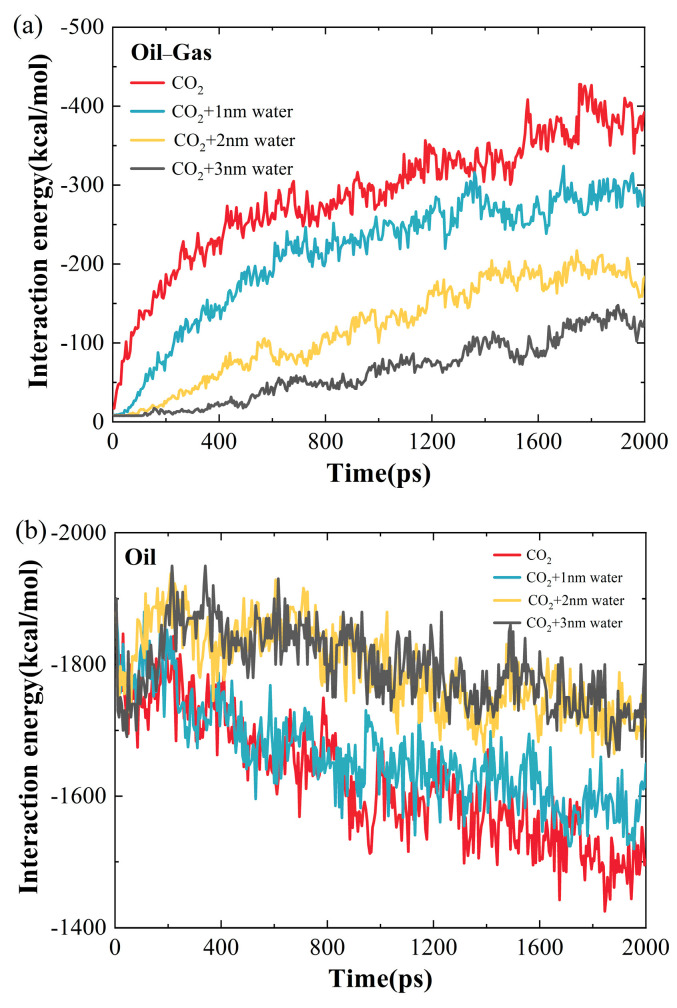

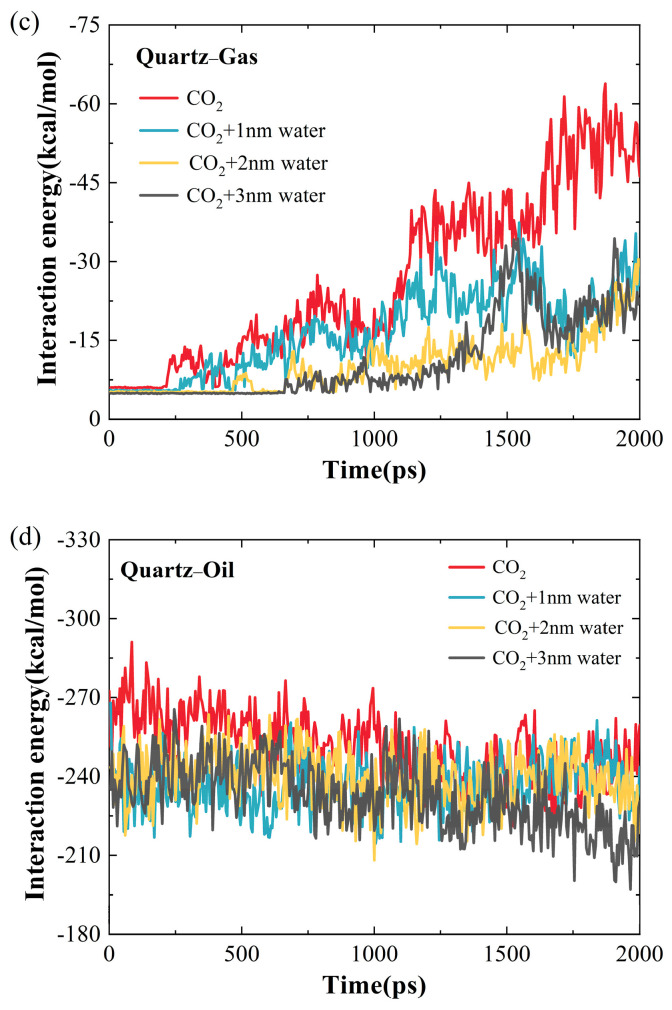
The interaction energy between quartz, gas, and crude oil under water conditions: (**a**) crude oil–gas; (**b**) crude oil–crude oil; (**c**) quartz–gas; (**d**) quartz–crude oil.

Figure 5 shows the interaction energies between different components varying with time across various water-film thicknesses. One can observe from Figure 5a that the magnitude of the interaction energy between oil and gas decreases with the increasing water-film thickness. Compared to the system without a water film, the interaction energies between crude oil and gas decrease by 25.78%, 58.13%, and 77.49% for the systems with water-film thicknesses of 1 nm, 2 nm, and 3 nm, respectively. This indicates that the water film impedes the movement of CO_2_ molecules toward the quartz wall, thereby weakening the association between CO_2_ and crude oil. In contrast, Figure 5b shows that the absolute value of the interaction energy between crude-oil molecules increases as the water film becomes thicker. Relative to the system without a water film, the crude-oil–crude-oil interaction energy increases by 2.41%, 10.45%, and 10.62% for the systems with water-film thicknesses of 1 nm, 2 nm, and 3 nm, respectively. The increase in crude-oil–crude-oil interaction energy is attributed to the reduced ability of CO_2_ to diffuse into the oil, which leads to a diminished capacity to reduce viscosity. Figure 5c shows that the magnitude of the interaction energy between quartz and CO_2_ gas also decreases with increasing water-film thickness. Specifically, when compared to the system without a water film, the interaction energy decreases by 37.56%, 57.76%, and 61.69% for the systems with water-film thicknesses of 1 nm, 2 nm, and 3 nm, respectively. This trend is due to the water film acting as a barrier to CO_2_ diffusion toward both the oil and the quartz, thereby lowering the probability of CO_2_ being adsorbed to the wall. Figure 5d presents the interaction energy between crude oil and the quartz surface, indicating that the effect of the water-layer thickness on the interaction energy is comparatively slight. However, a slight increase in the interaction energy is observed with increasing water-film thickness. This is probably due to the thicker water film acting as a barrier that limits CO_2_ infiltration into the crude oil, thereby alleviating the CO_2_-driven reduction in crude-oil density and slightly improving oil–quartz interactions.

Through quantitative analysis of radial distribution functions (Figure 3) and interaction energy (Figure 5), we found that the presence of water may give rise to a complex three-phase competitive interface. At this interface, CO_2_ molecules first interact with the water layer and must overcome its barrier. This process is strictly constrained by the CO_2_–water interaction energy and, in doing so, would consume part of the initial driving force for CO_2_ migration into the oil phase. Therefore, as the thickness of the water film increases, the interaction energy between CO_2_ and the oil phase decreases significantly, which may be not only due to the increase in mass transfer distance but also due to the complex three-phase interface behavior. This finding shows that the existence of the water film is not a simple physical barrier, and the water–CO_2_ interface interaction is also the controlling factor that determines the efficiency of CO_2_–oil displacement.

The above results discuss the microscopic processes of CO_2_–crude-oil interactions across varying water-film thicknesses. In practical production, temperature and pressure are also major factors affecting the CO_2_-EOR process [20]. Therefore, the subsequent section will examine a system with a 2 nm water film to assess the influence of temperature and pressure on the processes.

### 2.2. Effect of Temperature

**Figure 6:**
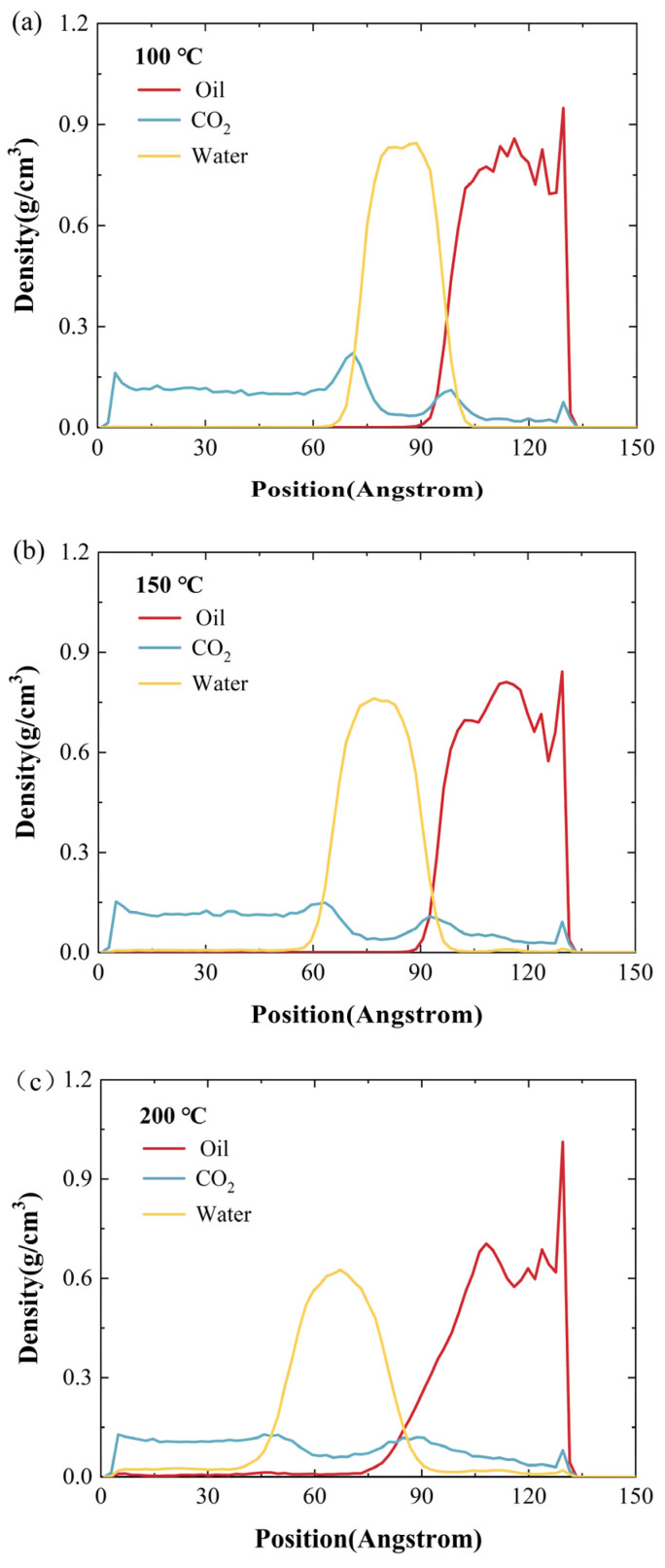

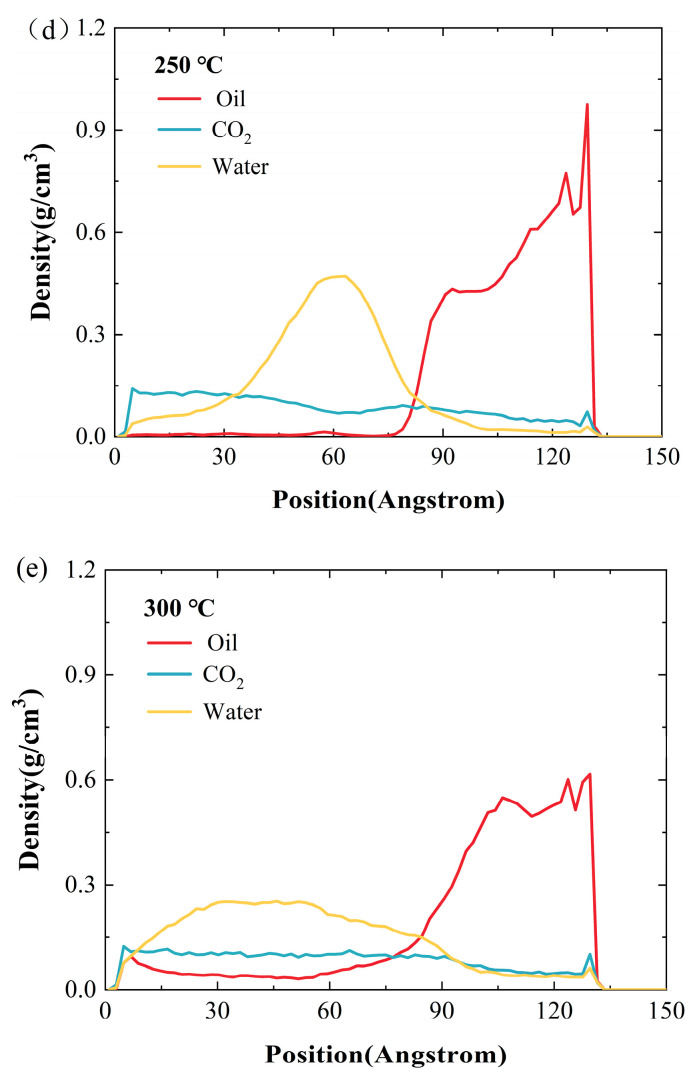
Density distribution of each component on the quartz wall at various temperatures: (**a**) 100 °C; (**b**) 150 °C; (**c**) 200 °C; (**d**) 250 °C; (**e**) 300 °C.

Figure 6 shows the density distribution characteristics of CO_2_, water, and crude oil at different temperatures (100 °C, 150 °C, 200 °C, 250 °C, 300 °C) under the conditions of a 10 MPa constant pressure and 2 nm water-film thickness. As is shown in Figure 6a, at 100 °C, the density of crude oil is 0.81 g/cm^3^, and the maximum density of the water film reaches 0.847 g/cm^3^. Currently, the interface of the oil–water two-phase structure is clear. As the temperature rises to 150 °C (Figure 6b), the densities of the crude-oil and water phases begin to decrease, indicating that CO_2_ molecules penetrate into water and crude oil. As the temperature continues to rise to 200 °C (Figure 6c), the densities of the oil phase and water phase continue to decrease, which means that the increase in temperature further loosens the structure of water and crude oil. At 250 °C (Figure 6d), the dispersion of CO_2_ in the water phase and oil phase is significantly intensified, and the peak density of the water film and oil phase both show a significant downward trend. When the temperature rises to 300 °C (Figure 6e), the density of crude oil drops to 0.55 g/cm^3^ (a decrease of 32.1%), and the density of the water film drops to 0.274 g/cm^3^ (a decrease of 66.67%). In addition, the peak value of the water phase density shifts to the CO_2_ region, indicating that many water molecules dissolve into the surrounding CO_2_ atmosphere. It is worth noting that with the increase in temperature, the enrichment of CO_2_ at the oil–water interface gradually decreases, and at 300 °C, the accumulation of CO_2_ at the interface almost disappears, which indicates that the interfacial tension is greatly reduced and the miscibility of the three components of CO_2_, water, and crude oil is significantly improved. These temperature-dependent density-curve-evolution laws fully confirm that the molecular mixing process under high-temperature environments can effectively promote the penetration of CO_2_ into crude oil, which has a significant positive effect on improving the oil recovery rate of water-bearing reservoirs.

**Figure 7:**
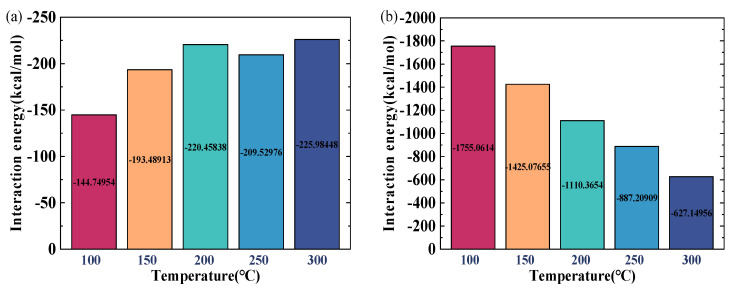

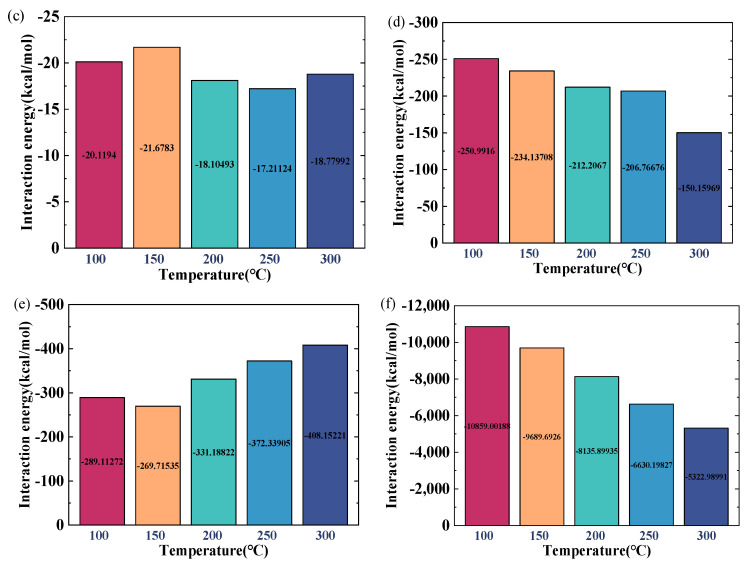
Interaction energy between components at various temperatures: (**a**) oil–CO_2_; (**b**) oil–oil; (**c**) quartz–CO_2_; (**d**) quartz–oil; (**e**) water–CO_2_; (**f**) water–water.

Figure 7 illustrates the interaction energies among various components under different temperature conditions. The interaction energies presented in this paper were calculated as the average values obtained from the last 500 picoseconds (ps) of the simulation. As is shown in Figure 7a, the interaction energy between crude oil and gas increases by 36.28% when the temperature rises from 100 °C to 300 °C, while the interaction energy between crude-oil molecules decreases by 64.27% (Figure 7b), indicating a reduction in intermolecular attraction that facilitates a viscosity reduction. The interaction energy between CO_2_ and the quartz wall (Figure 7c) first increases and then decreases with temperature elevation: initially, higher temperatures promote more CO_2_ molecules to penetrate into crude oil and adsorb onto the quartz wall, enhancing the interaction, but further temperature increases enhance the CO_2_ molecular mobility, causing the molecules to permeate into the oil and water phases and reducing accumulation near the quartz surface. The interaction energy between crude oil and the quartz surface (Figure 7d) decreases by 40.24% over the same temperature range, as strengthened thermal motion drives crude-oil molecules away from the wall, weakening adsorption and promoting oil detachment. Meanwhile, the interaction between gas and the water film (Figure 7e) increases with temperature due to enhanced water molecular activity, which expands the water film and increases intermolecular spacing, facilitating CO_2_ transfer into the water layer and boosting mixing; this effect also reduces water–water interaction energy.

The reduction in water–water interaction energy, as shown in Figure 7f, directly indicates that the stability of the water-film barrier is greatly reduced. The reason for this is that the increase in temperature causes the kinetic energy of water molecules to increase significantly, thereby destroying the strong hydrogen bond network that maintains the close arrangement of water molecules, causing the water-layer structure to become more disordered and the permeability to increase significantly. The structural integrity of the water film degrades due to the increase in temperature, which creates favorable conditions for CO_2_ penetration and significantly enhances the mixing effect of CO_2_ and crude oil. These findings suggest that the effect of temperature on the system may not be limited to acting on the oil phase and CO_2_ phase alone but also fundamentally reshapes the properties of the water phase. Under low-temperature conditions, the water phase acts as a strong barrier medium, limiting the interaction between CO_2_ and crude oil, while in a high-temperature environment, the water phase is transformed into a highly permeable medium, which helps the migration and diffusion of CO_2_.

### 2.3. Effect of Pressure

**Figure 8:**
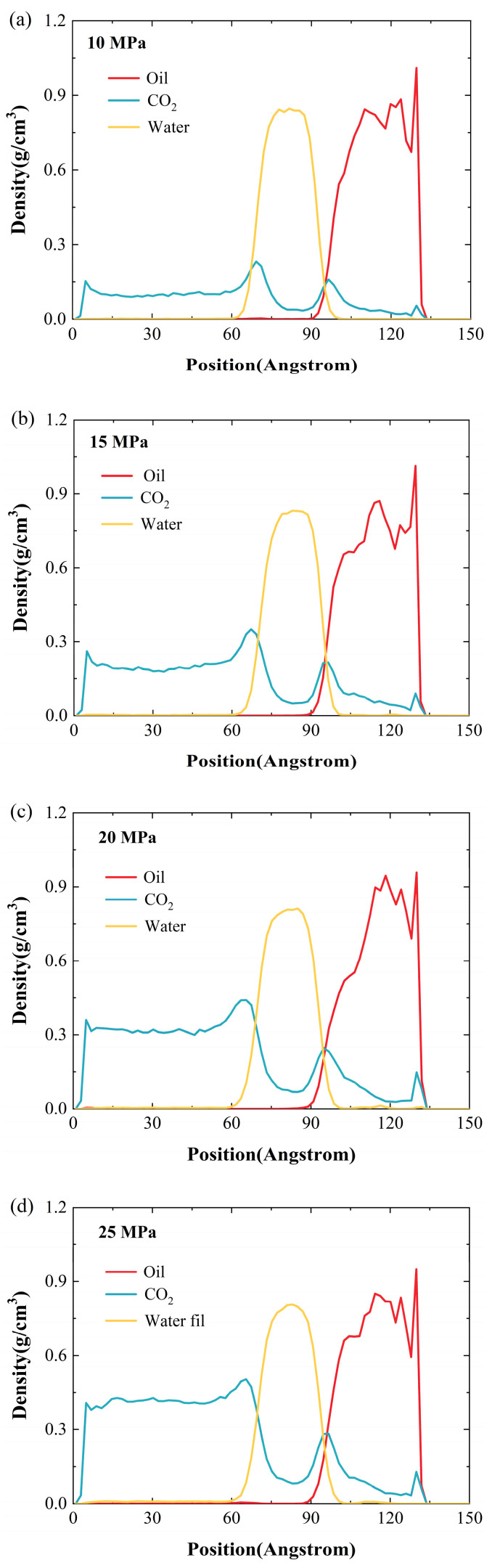

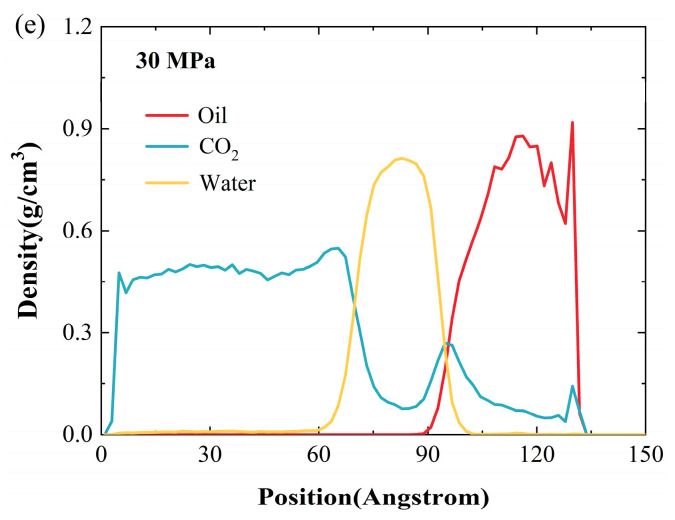
Density profiles of oil and gas on the quartz wall under various pressures: (**a**) 10 MPa; (**b**) 15 MPa; (**c**) 20 MPa; (**d**) 25 MPa; (**e**) 30 MPa.

Figure 8 shows the density distribution of oil, CO_2_, and water under different pressures (10, 15, 20, 25, and 30 MPa) at 100 °C and a water-film thickness of 2 nm. At 10 MPa (Figure 8a), the density curves of oil and CO_2_ are clearly separated, and the density of CO_2_ near the oil phase is very low, indicating that the solubility is limited and the interaction between the two is weak. When the pressure rises to 15 MPa (Figure 8b), the density of CO_2_ near the oil phase increases slightly, and the distribution of oil begins to become more diffuse. At 20 MPa (Figure 8c), the CO_2_ density near the quartz wall continues to climb, and the oil-phase density peak begins to move slightly away from the wall, which means that the interaction between CO_2_ and oil is enhanced. As the pressure further increases to 25 MPa and 30 MPa, a dense CO_2_ layer is formed at the wall surface, as shown in Figure 8d,e, and some oil molecules adsorbed on the quartz surface are effectively replaced by CO_2_. Although the density changes in the water film and the oil phase were not as significant as the effect caused by the temperature change over the entire pressure range, their density peaks still decreased by 5.88% and 4.39%, respectively. This density reduction phenomenon is attributed to the increase in the solubility of CO_2_ in the water phase and the oil phase as the pressure increases.

**Figure 9:**
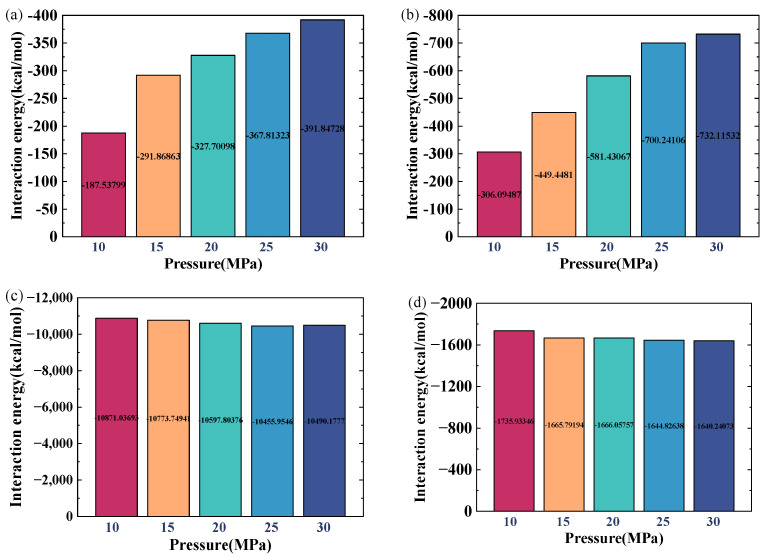

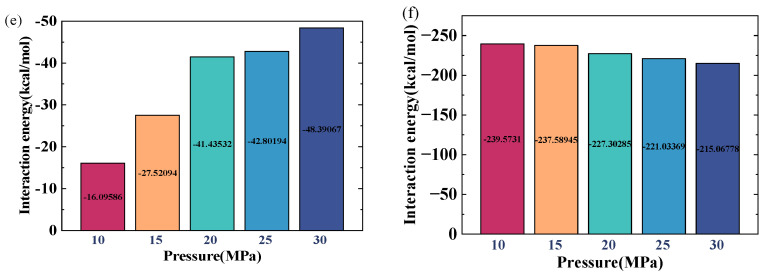
Interaction energies between components at different pressures: (**a**) oil–gas; (**b**) water-film–gas; (**c**) water–water; (**d**) oil–oil; (**e**) quartz–gas; (**f**) quartz–oil.

Figure 9 presents the interaction energies between various components under different pressures. When the gas pressure increases from 10 MPa to 30 MPa, the interaction energy between crude oil and gas rises by 52.17%, and that between the water film and gas increases by 58.19% (Figure 9a,b), mainly due to the enhanced diffusion of CO_2_ molecules into the oil and water film under higher pressures. Correspondingly, the interaction energies between water–water molecules and between crude-oil molecules (Figure 9c,d) decrease by 3.50% and 5.48%, respectively. The interaction energy between CO_2_ and the quartz surface shows a 66.74% increase (Figure 9e) as elevated pressures lead to more CO_2_ molecules accumulating and adsorbing near the quartz surface. In contrast, Figure 9f reveals that the interaction energy between quartz and crude oil drops by 10.23% with increasing pressure, as more CO_2_ molecules dissolve into the crude oil and attach to the quartz wall, weakening the direct interaction between quartz and crude oil, and thus facilitating the separation of crude-oil molecules from the quartz interface.

### 2.4. Practical Implications for CO_2_-EOR

This study focuses on the microscopic mechanisms controlling the behavior of CO_2_ molecules and their interactions under the coexistence conditions of water, oil, and rock surfaces. However, these in-depth molecular-level insights are of crucial relevance for understanding and optimizing the operation of CO_2_ enhanced oil recovery (CO_2_-EOR) at the macroscopic scale [21,22]. For CO_2_-EOR, its key effects such as the oil extraction efficiency and the reduction in crude-oil viscosity fundamentally depend on whether the injected CO_2_ can effectively contact and fully dissolve in the crude oil, thereby achieving volume expansion, viscosity reduction, and component extraction. Through microscopic simulation, we have elaborated in detail upon the key barrier role of the water film regarding the diffusion of CO_2_: The existence of the water film significantly hinders the direct contact and effective transfer between CO_2_ molecules and crude oil, thereby limiting the dissolution of CO_2_ and the subsequent improvement of crude-oil properties. Furthermore, this research has clearly revealed how temperature and pressure conditions affect the interaction mechanism of this CO_2_–water–oil interface and their regulatory role in CO_2_ transfer. These mechanistic findings on the microscopic behavior of CO_2_ in multiphase and nano-pores directly point to the core factors that limit the displacement efficiency of CO_2_ at the microscopic scale. These results indicate that if this physical barrier formed by water films is widespread in the vast pore network of the oil reservoir, it will inevitably be manifested at the on-site macroscopic scale as an insufficient CO_2_ ripple volume with reduced ripple efficiency, and ultimately lead to a decline in the overall crude-oil recovery rate, etc.

Therefore, the microscopic mechanism of the water film hindering CO_2_ transfer revealed at the molecular level in this study, as well as the specific laws of these phenomena under different temperature and pressure conditions, can provide a crucial basic scientific basis for the implementation and optimization of macroscopic CO_2_-EOR projects.

## 3. Methodology and Analysis

### 3.1. Molecular Model Construction and Validation

**Figure 10:**
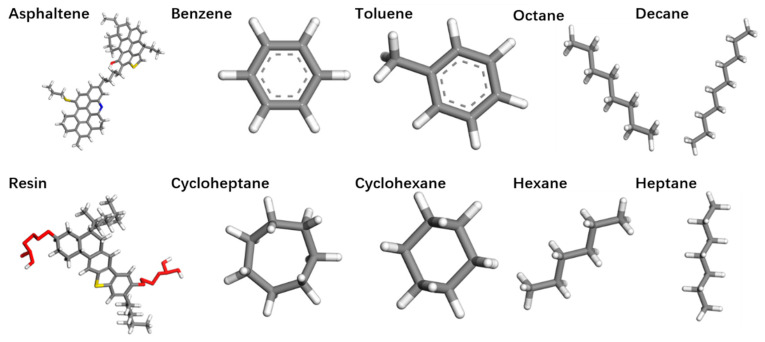
Molecular models of the four components in crude oil (gray represents carbon atoms, white indicates hydrogen atoms, red signifies oxygen atoms, blue denotes nitrogen atoms, and yellow corresponds to sulfur atoms).

Crude oil is generally a multi-component mixture, so when modeling crude oil, the influence of different components must be considered. According to the classic four-component separation (SARA) method, crude oil is typically divided into four components: saturates, aromatics, resins, and asphaltenes [23,24]. Among these, the large molecular components in crude oil, such as asphaltenes and resins, significantly affect the density and dynamic properties of the crude oil. Therefore, these large molecular components cannot be ignored in the construction of crude-oil models. During the modeling procedures employed in this research, asphaltenes, resins, and aromatics (benzene and toluene), along with saturates (cycloheptane, cyclohexane, decane, heptane, hexane, and octane), are selected as crude-oil components. The molecular models of each component are shown in Figure 10. In this study, the modeling of asphaltenes is based on the average molecular model of asphaltenes, namely the at−N +2S +O asphaltene model and the modified R-benzo-thiophene-S molecular model [25].

To verify the accuracy of the models described above, the densities of the individual molecular models were first calculated and compared with available experimental results and data from the NIST database. For the saturates and aromatics, typical models of C_6_ and toluene were used for validation. The specific calculation process is as follows: The systems of the four components were constructed using an amorphous cell and subjected to geometric optimization and energy minimization. Then, the density of the simulated system was calculated using the Forcite dynamics module. During the model validation process, the simulation parameters were as follows: ensemble (NPT), force field (COMPASS), temperature (293.15 K), and pressure (0.1 MPa). The Ewald and atom-based summation techniques were employed to characterize electrostatic and van der Waals interactions. The cutoff radius for the van der Waals interactions is 1.25 nm. The Andersen approach was utilized to regulate temperature, while the Berendsen method was employed to maintain pressure.

**Figure 11:**
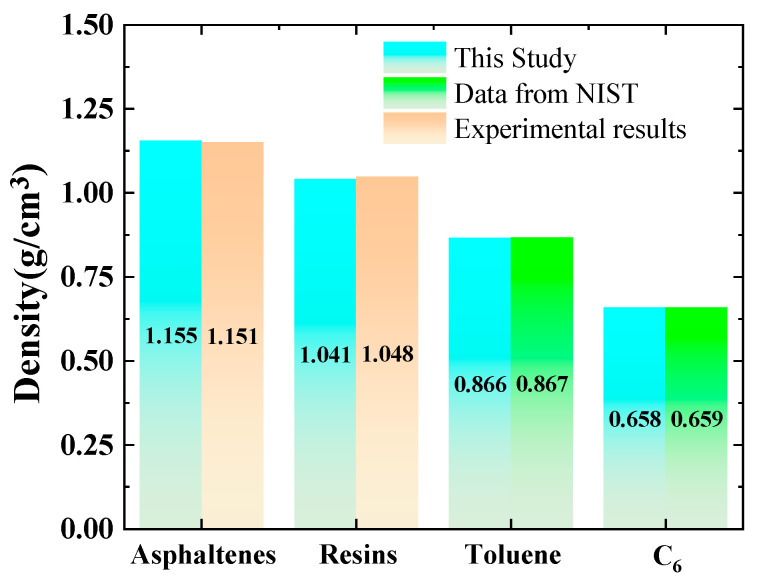
Comparison of the calculated densities for different crude-oil-component by molecular models with the data from NIST and experimental data [25].

Figure 11 shows the comparison of densities for different crude-oil components with experimental and NIST results. As is seen in this figure, the simulated densities for asphaltenes and resins match well with the computed and experimental values provided by Li et al. [25]. The calculated densities of C_6_ and toluene are consistent with the values in the NIST database, and the density errors of all components are less than 3%. Therefore, it can be considered that the constructed molecular model of crude-oil components is reasonable and can be used for subsequent calculation and analysis.

**Figure 12:**
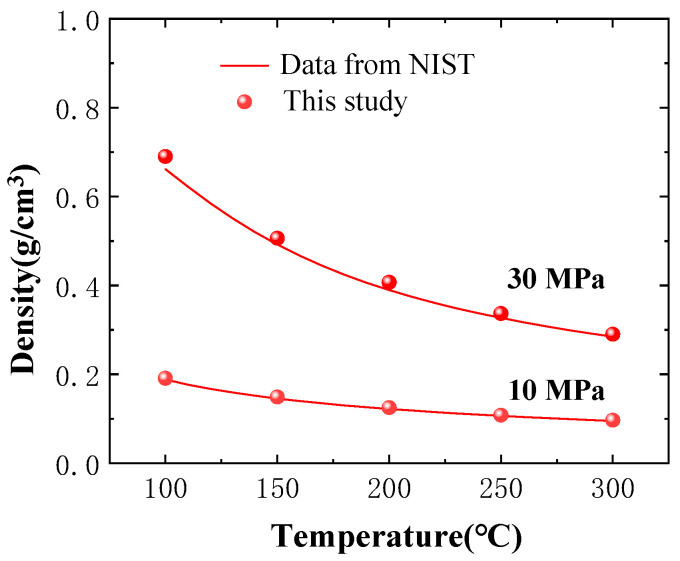
Density validation of the CO_2_ molecular model.

Furthermore, to confirm the validity of the MD method in simulating gas properties, as depicted in Figure 12, the density of CO_2_ gas at various pressures and temperatures was computed and then compared to the values in the NIST database. It can be observed that the density of CO_2_ matches well with the values from the NIST database, indicating that the construction of the gas molecular model, as well as the computational methods and parameter settings in this study, are reasonable.

### 3.2. Construction of the Water–Crude-Oil–Rock-Wall Model

**Figure 13:**
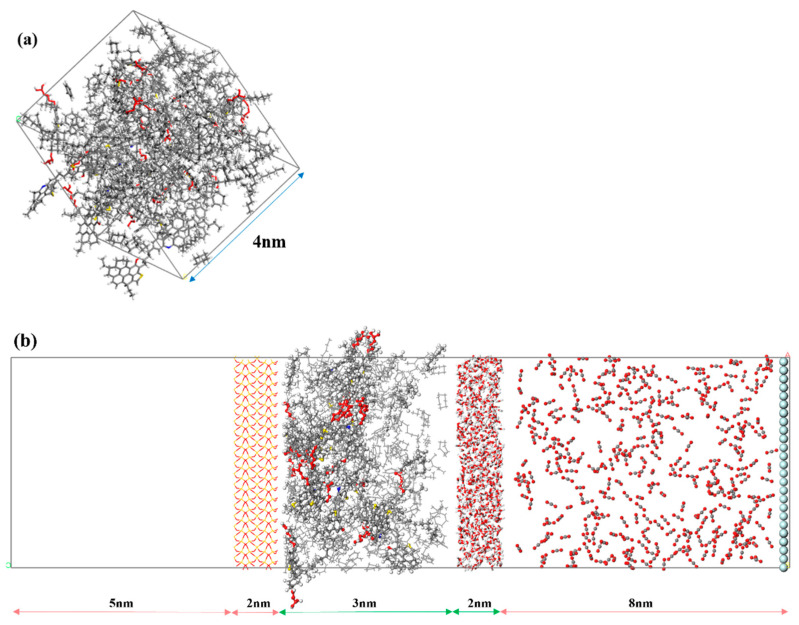
Schematic diagram of the model: (**a**) crude-oil model; (**b**) gas–water–crude-oil–rock model.

To study how the water film impacts gas dissolution, diffusion within crude oil, and its interaction with the crude-oil matrix, a molecular assembly featuring CO_2_, a water film, crude oil, and a rock wall was constructed, as shown in Figure 13. The process of model construction is described in detail below:

**(1) Construction of the rock-wall surface**: Quartz is the most common silicon oxide compound and a typical non-clay inorganic mineral, forming the main component of rock-wall surfaces. In this study, the α-quartz crystal structure developed by Will et al. [26] was selected, with the commonly used (0 0 1) crystal plane chosen as the wall interacting with the fluid. The size of the quartz crystal surface is 49.1 Å × 42.5 Å.

**(2) Construction of the crude-oil model**: Crude oil is a mixture of hydrocarbons and other compounds. In this section, the model of crude oil is constructed using components, including asphaltenes, resins, saturates, aromatics, etc., as listed in Table 1. The relative proportions of these SARA fractions were precisely determined to reflect the experimental characterization data of typical crude oil in references [27,28]. Specifically, the component ratios include an asphaltene mass fraction of 13.3%, a resin mass fraction of 24.8%, a saturated substance mass fraction of 51.2%, and an aromatic hydrocarbon mass fraction of 10.7%. By using this model, the simulated crude oil is a physically representative system rather than merely a random mixture. The dimensions of the crude-oil model are 30.0 × 49.0 × 42.0 Å, as shown in Figure 13a.

**(3) Construction of the gas–water–crude-oil–rock model**: A water layer of a certain thickness, a 3 nm crude-oil layer, and the previously constructed rock-wall surface were combined, followed by a 1 ns simulation to stabilize the oil adsorption layer on the rock surface. Then, this was combined with a gas box of a certain density to form the gas–water–crude-oil–rock model. In addition, to ensure that the gas molecules on the right side of the model did not diffuse to the left side of the rock wall, a layer of He atoms was fixed on the far right of the model. To minimize the influence of the rock wall on the system, a 5 nm vacuum layer was added to the left side of the rock, as shown in Figure 13b.

### 3.3. Simulation Details

The specific simulation details are as follows: The NVT ensemble [29] and the COMPASS force field [30,31] were used in the simulation. This force field allows for the detailed prediction of molecular configurations and their energetic characteristics of most molecules. The temperature controller was set to Andersen, the van der Waals interactions were set to be atom based, and the electrostatic interactions were described using the Ewald method. A time increment of 1.0 fs was used.

## 4. Conclusions

In this study a molecular model of CO_2_–water-film–oil–rock in water-bearing reservoirs was developed, and the influence of water-film thickness on the CO_2_–crude-oil interaction was investigated by using MD simulations. In addition, the impacts of reservoir temperature and pressure were also analyzed. The main outcomes are outlined below:

(1) The water film can impede the migration of CO_2_ molecules into the oil, resulting in a reduced ability of CO_2_ to lower the oil density, and this impact becomes more significant as the thickness of the water film increases. As the thickness of the water film increases from 0 nm to 3 nm, the density of oil increases by 86.9%. The water film also hinders the diffusion ability of oil molecules. When the water-film thickness reaches 3 nm, the diffusion coefficient of oil drops by 72.30% relative to a system lacking a water layer.
(2) A rise in temperature significantly improves the ability of CO_2_ and water to diffuse into oil, thereby reducing the oil’s density. With the pressure of 10 MPa and a 2 nm water film, as the temperature rises from 100 °C to 300 °C, the density of crude oil declines by 32.1%.
(3) Increasing the reservoir pressure results in a decline in the density of crude oil as well as the water film in the water-bearing reservoir. However, the effect of pressure is relatively small compared to the temperature change. At a temperature of 100 °C and a water-film thickness of 2 nm, the oil density decreases by only 4.39% when the pressure increases from 10 MPa to 30 MPa.

This study employed molecular dynamics to investigate the effects of water-film thickness, temperature, and pressure on CO_2_–oil–rock interactions. The results provide molecular-level insights that can guide gas-injection strategies in water-bearing reservoirs. However, the dynamic displacement process was not considered and will be addressed in future work.

## Figures and Tables

**Table 1:** Crude-oil component list.

Substance	Number	Mass Fraction (%)
Average Asphaltenes	5	13.3
Average Resins	11	24.8
Hexane	26	6.9
Heptane	24	7.4
Octane	28	9.9
Decane	32	14.1
Cyclohexane	17	4.4
Cycloheptane	28	8.5
Benzene	11	2.7
Toluene	28	8

## Data Availability

The data presented in this study are available on request from the corresponding author.
